# Clinical Characteristics and Prognosis of Esophageal Squamous Cell Carcinoma in Patients Under 60 Years of Age

**DOI:** 10.3390/cancers17223642

**Published:** 2025-11-13

**Authors:** Dae-Gon Ryu, Cheol-Woong Choi, Su-Jin Kim, Su-Bum Park, Jin-Ook Jang, Woo-Jin Kim, Cheol-Min Lee, Soo-Bin Synn, Eun-Jung Choi, Bong-Soo Son, Sun-Hwi Hwang, Si-Hak Lee, Jae-Hun Chung

**Affiliations:** 1 Department of Internal Medicine, Pusan National University School of Medicine, Research Institute for Convergence of Biomedical Science and Technology, Pusan National University Yangsan Hospital, Yangsan 50612, Republic of Korea; gon32gon@pusan.ac.kr (D.-G.R.); a_warm_mind@naver.com (J.-O.J.);; 2 Department of Thoracic and Cardiovascular Surgery, Pusan National University School of Medicine, Research Institute for Convergence of Biomedical Science and Technology, Pusan National University Yangsan Hospital, Yangsan 50612, Republic of Korea; 3 Department of Surgery, Pusan National University School of Medicine, Research Institute for Convergence of Biomedical Science and Technology, Pusan National University Yangsan Hospital, Yangsan 50162, Republic of Koreaghost109@hanmail.net (S.-H.L.);

**Keywords:** esophageal cancer, squamous cell carcinoma, age

## Abstract

**Background:** Esophageal squamous cell carcinoma (ESCC) typically occurs in older individuals. The etiology and clinical characteristics of ESCC in relatively younger patients under 60 years of age remain unclear. Understanding whether age affects tumor behavior or prognosis is important for improving patient management. This study aimed to analyze the characteristics of ESCC diagnosed before the age of 60. **Methods:** We retrospectively reviewed the medical records of ESCC patients diagnosed between December 2008 and May 2025. A total of 516 patients were divided into two groups based on whether they were aged 60 or above. Medical history, clinical features, and outcomes were compared between the two groups. **Results:** There were 100 patients under 60 years and 416 patients aged 60 and above. The median ages were 55 (range 41–59) and 72 (range 60–95), respectively. Younger patients had a significantly stronger association with heavy drinking (72.0% vs. 39.2%, *p* < 0.001) and smoking (76.0% vs. 55.0%, *p* < 0.001). There was a trend toward more advanced disease (Stage IV: 26.0% vs. 18.5%, *p* = 0.094) and metastatic presentation (18.0% vs. 13.9%, *p* = 0.305) in the under-60 group. Despite being younger with better performance status and fewer comorbidities, their overall survival did not differ from that of older patients (HR 0.92; 95% CI, 0.67–1.26; *p* = 0.593). **Conclusions:** Patients diagnosed with ESCC under the age of 60 showed a stronger association with heavy alcohol consumption and smoking and more frequent presentation with advanced-stage disease compared with older patients. Younger age did not confer a better prognosis, highlighting the importance of early detection and timely intervention regardless of patient age.

## Simple Summary

Esophageal squamous cell carcinoma (ESCC) usually occurs in older adults, but it can also affect younger individuals. However, little is known about whether younger patients show different clinical characteristics or outcomes compared with older ones. In this study, we analyzed 516 patients diagnosed with ESCC and compared those younger than 60 years with those aged 60 years or older. We found that younger patients were more likely to have a history of heavy alcohol consumption and smoking and tended to present with more advanced-stage disease at diagnosis. Being younger did not translate into a better prognosis—the overall survival was similar between the two age groups. These findings suggest that age alone may not determine prognosis in ESCC and that careful evaluation and timely treatment are equally important for both younger and older patients.

## 1. Introduction

Esophageal cancer is the 8th most diagnosed cancer worldwide and ranks as the 6th leading cause of cancer-related death globally [1]. Among its histologic subtypes, esophageal squamous cell carcinoma (ESCC) is the predominant type, particularly in East Asian countries such as Korea, China, and Japan, where the incidence and mortality rates are disproportionately high [2,3,4]. The major risk factors for ESCC include heavy alcohol consumption and tobacco smoking, which act synergistically to promote carcinogenesis in the esophageal epithelium [5,6,7,8]. In East Asian populations, a common genetic variant of aldehyde dehydrogenase 2 has been associated with alcohol-related ESCC due to impaired acetaldehyde metabolism [9].

ESCC occurs predominantly in the elderly, and with the global increase in life expectancy, the prevalence of ESCC is rising steadily [2,3]. Recent epidemiological data indicate that the peak incidence of ESCC occurs in the seventh decade of life, underscoring its strong association with advanced age [3,10]. Consequently, ESCC in relatively younger patients under the age of 60 is uncommon, and limited studies have investigated its clinical and pathological characteristics. However, recent reports suggest that the proportion of early-onset ESCC cases has gradually increased, possibly due to lifestyle factors such as early initiation of alcohol consumption and smoking [11,12]. Moreover, several studies have indicated that younger-onset cancer may present with more advanced disease at diagnosis, underscoring the importance of understanding its distinct clinical features [13,14,15]. This study aimed to explore the etiologic factors, clinical features, and prognostic outcomes of ESCC in patients younger than 60 years.

## 2. Material and Methods

### 2.1. Patients

The medical records of individuals diagnosed with esophageal cancer between December 2008 and May 2025 at Pusan National University Yangsan Hospital (South Korea) were retrospectively reviewed. Among those with a histopathologic diagnosis of esophageal squamous cell carcinoma (ESCC), patients lacking definitive staging information or follow-up data were excluded. In total, 516 eligible patients were included in the final analysis. For comparison, the study population was stratified into two groups according to age: those under 60 years (*n* = 100) and those aged 60 years or older (*n* = 416). The patients’ medical history, including alcohol consumption, smoking status, and history of surgery, was investigated. Heavy drinking was defined according to the criteria of the National Institute on Alcohol Abuse and Alcoholism as consuming more than 14 standard drinks per week for men and more than 7 drinks per week for women [16]. The Eastern Cooperative Oncology Group (ECOG) performance status was used to assess patients’ general functional status, ranging from 0 (fully active) to 4 (completely disabled). The Charlson Comorbidity Index (CCI) was calculated for each patient to quantify comorbidity burden and predict overall mortality risk based on weighted comorbid conditions [17].

This study received approval from the Institutional Review Board (IRB) of Pusan National University Yangsan Hospital (approval no. 55-2025-095). The need for informed consent was waived by the IRB because all patient data were anonymized prior to analysis. All study procedures were carried out in compliance with institutional ethical standards and relevant national regulations.

### 2.2. Staging Workup and Treatment Dicision

All patients included in this study were histologically diagnosed with ESCC based on endoscopic biopsy before staging evaluation. Before initiating treatment, all patients underwent a comprehensive staging evaluation that included upper gastrointestinal endoscopy, endoscopic ultrasound (EUS), chest and abdominal computed tomography (CT), and 18F-fluorodeoxyglucose positron emission tomography/computed tomography (FDG-PET/CT). The depth of tumor invasion (T stage) was primarily assessed by EUS, while involvement of adjacent structures was verified using both EUS and CT imaging. Regional lymph node metastases were evaluated through combined findings from EUS, CT, and FDG-PET/CT, and distant metastases were identified based on CT and PET/CT results. The final disease stage was determined according to the 8th edition of the American Joint Committee on Cancer staging system [18]. Treatment decisions were made through multidisciplinary tumor board discussions, and therapeutic strategies were generally based on the Japan Esophageal Society practice guidelines available at the time of treatment (2007, 2012, 2017, and 2022 editions) [19,20,21,22]. In some cases, no cancer-specific treatment was performed when patients had extremely poor general condition or declined active therapy.

### 2.3. Surgery

Patients who underwent surgical treatment received an Ivor Lewis esophagectomy with intrathoracic anastomosis and either two-field or three-field lymphadenectomy. In cases with prior anatomical alterations—such as a history of gastrectomy—reconstruction was performed using a jejunal or colonic interposition graft. One patient with cervical ESCC underwent combined esophagectomy and laryngectomy. Circular stapled anastomosis was used in all operations. The integrity of the anastomosis was evaluated approximately one week postoperatively by esophagography and endoscopic examination. Pathological assessment was performed to determine margin status (R0 resection) and to confirm the presence or absence of lymph node metastasis.

### 2.4. Chemoradiotherapy

Chemoradiotherapy (CRT) was classified as either definitive CRT, administered with curative intent, or neoadjuvant CRT delivered before surgery. According to each patient’s clinical status, some individuals received definitive radiation therapy (RT), whereas others underwent palliative RT solely for symptomatic management.

Radiation treatment was performed using either three-dimensional conformal radiotherapy or intensity-modulated radiotherapy techniques. The prescribed RT schedules were as follows: (1) definitive CRT, 2 Gy per fraction for a total of 50 Gy (25 fractions); (2) neoadjuvant CRT, 1.8 Gy per fraction for a total of 45 Gy (25 fractions); and (3) palliative RT, 3 Gy per fraction for a total of 30 Gy (10 fractions). Chemotherapy regimens primarily included either cisplatin plus 5-fluorouracil (cisplatin 75 mg/m^2^ on day 1 and 5-fluorouracil 750 mg/m^2^/day on days 1–4 during weeks 1 and 5) or paclitaxel plus carboplatin (paclitaxel 50 mg/m^2^ and carboplatin at an area under the curve of 2 mg/mL/min, administered weekly for five weeks).

### 2.5. Endoscopic Resection

Endoscopic resection was performed in patients with superficial esophageal carcinoma that showed no evidence of lymph node metastasis on preoperative imaging and appeared confined to the mucosal layer on endoscopic ultrasound. Most of these patients underwent endoscopic submucosal dissection (ESD), which was carried out under conscious sedation using intravenous midazolam, without the need for general anesthesia. In the absence of procedure-related complications, patients were typically discharged approximately two days after treatment. Complete resection was defined as en bloc removal of the lesion with histologically tumor-free margins. Curative resection was defined as complete resection without submucosal invasion, lymphovascular infiltration, or poorly differentiated histologic features.

### 2.6. Follow-Up After Treatment

Following curative treatment—including surgery, definitive CRT, or endoscopic resection—patients underwent surveillance with endoscopy and chest/abdominal CT approximately 2–3 months after therapy, every 3 months during the first year, and every 6 months thereafter. PET was not routinely performed after surgery or endoscopic resection but was obtained 3–6 months after completion of definitive CRT. Surgical resection was generally scheduled within 2 months after neoadjuvant CRT; however, some patients were managed non-surgically based on multidisciplinary review and preoperative findings. A clinical complete response after CRT was defined as the absence of detectable tumor on endoscopy, biopsy, CT, and PET performed 2–3 months post-treatment. When post-CRT inflammation or ulceration made interpretation difficult, these evaluations were repeated after an additional 2–3 months.

### 2.7. Statistical Analysis

Categorical variables were summarized as numbers and percentages, whereas continuous variables were expressed as medians with corresponding ranges. Differences between groups were assessed using Fisher’s exact test for categorical variables and Student’s *t*-test for continuous variables. Follow-up time was reported as the median duration (range) in months. Survival probabilities were estimated using the Kaplan–Meier method, and comparisons between survival curves were evaluated with the log-rank test. A Cox regression model was used to estimate the HR and 95% CI associated with survival. A *p* value < 0.05 was considered statistically significant. The Statistical Package for the Social Sciences version 27.0 (IBM Corp., Armonk, NY, USA) was used for statistical analyses.

## 3. Results

### 3.1. Baseline Characteristics of All Patients

During the study period, a total of 516 patients with histologically confirmed ESCC were identified. The median follow-up duration was 19 months (range, 1–197). The median age at diagnosis was 69 years (range, 41–95), and 19.4% (*n* = 100) of the patients were under 60 years. Male patients accounted for 89.9% (*n* = 464) of the total cohort. A history of heavy alcohol consumption was noted in 45.5% (*n* = 235) of patients, and 59.7% (*n* = 308) had a history of smoking. Prior gastrectomy was reported in 5.4% (*n* = 28) of the patients, of whom 23 had undergone surgery for gastric cancer. The tumor was most commonly located in the mid-thoracic esophagus (45.5%, *n* = 235). Clinical stages ranged evenly from stage I to IV, with 14.7% (*n* = 76) presenting with distant metastasis at diagnosis. More than half of the patients (57.8%, *n* = 298) had an ECOG performance status of 0 or 1. The median CCI was 5 (range, 2–13). Surgery was the most frequently administered treatment modality (45.3%, *n* = 234), while 14.9% (*n* = 77) received no cancer-specific treatment. The baseline characteristics are summarized in Table 1.

### 3.2. Characteristics of ESCC Patients Under 60 Years of Age

The median age was 55 years (range, 41–59 years) in the <60-year group and 72 years (range, 60–95 years) in the ≥60-year group. A history of heavy alcohol consumption (72.0% vs. 39.2%, *p* < 0.001) and smoking (76.0% vs. 55.0%, *p* < 0.001) was significantly more common in patients under 60 years of age. Conversely, a history of gastrectomy was more frequently observed in patients aged 60 years or older (1.0% vs. 6.5%, *p* = 0.060), and the indication for gastrectomy was gastric cancer in 1 of 1 patient in the <60-year group and in 22 of 27 patients in the ≥60-year group (100% vs. 81.5%, *p* = 0.855). Although there was no statistically significant difference in stage distribution between the two groups, there was a trend toward a higher proportion of stage IV disease in younger patients (26.0% vs. 18.5%, *p* = 0.094). Similarly, the incidence of distant metastasis at diagnosis tended to be higher in the <60-year group (18.0% vs. 13.9%, *p* = 0.305). The proportion of patients with good performance status (ECOG 0 or 1) was significantly higher in the younger group (67.0% vs. 55.8%, *p* = 0.042), indicating better overall functional status. While most comorbid conditions were more common in the ≥60-year group, cirrhosis was the only condition found more frequently in younger patients (19.0% vs. 12.7%, *p* = 0.107). CCI was significantly lower in younger patients (median [range], 3 [2–11] vs. 5 [4–13], *p* < 0.001). Regarding treatment, surgical resection was performed more frequently in patients under 60 (60.0% vs. 41.8%, *p* = 0.001), whereas endoscopic resection was more commonly performed in older patients (6.0% vs. 16.3%, *p* = 0.011). Despite their younger age and relatively good general condition, 10.0% of patients under 60 years did not undergo any cancer treatment. A detailed comparison of clinical characteristics between the two age groups is summarized in Table 2.

### 3.3. Characteristics of Patients Who Underwent Surgery

Among the 234 patients who underwent surgical resection, 60 patients were under 60 years of age, and 174 patients were aged 60 years or older. In the younger group, all patients except one with a prior history of gastrectomy underwent esophago-gastric anastomosis. In contrast, among older patients, 10 had a history of gastrectomy; 11 patients underwent esophago-colonic anastomosis, and 3 patients underwent esophago-jejunal anastomosis. Neoadjuvant CRT was administered to 11 patients (18.3%) in the <60-year group and 40 patients (30.0%) in the ≥60-year group. Among those, pathologic complete remission was achieved in 3 patients (27.2%) and 12 patients (30.0%), respectively, with no significant difference between groups (*p* = 0.861). R0 resection was achieved in 53 patients (88.3%) and 156 patients (89.7%), respectively (*p* = 0.775). No significant difference was observed in the pathological stage after surgery between the two groups. Although postoperative mortality was lower in the younger group (3.3% vs. 8.6%), the difference was not statistically significant (*p* = 0.190). The rate of anastomotic leakage, a representative acute postoperative complication, was also lower in patients under 60 years (6.7% vs. 9.8%, *p* = 0.471), while the incidence of anastomotic stricture, a long-term complication, was higher in the younger group (20.0% vs. 13.2%, *p* = 0.207); however, both differences were not statistically significant. A summary of the comparison between the two groups of patients who underwent surgery is presented in Table 3.

### 3.4. Survival

**Figure 1:**
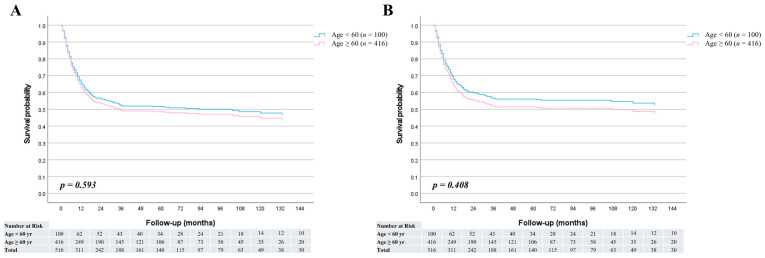
Survival curves comparing patients aged <60 and ≥60 years; (**A**) Overall survival was similar between the two groups (HR = 0.92; 95% CI, 0.67–1.26; *p* = 0.593). (**B**) Cancer-specific survival also showed no significant difference (HR = 0.87; 95% CI, 0.62–1.21; *p* = 0.408).

**Figure 2:**
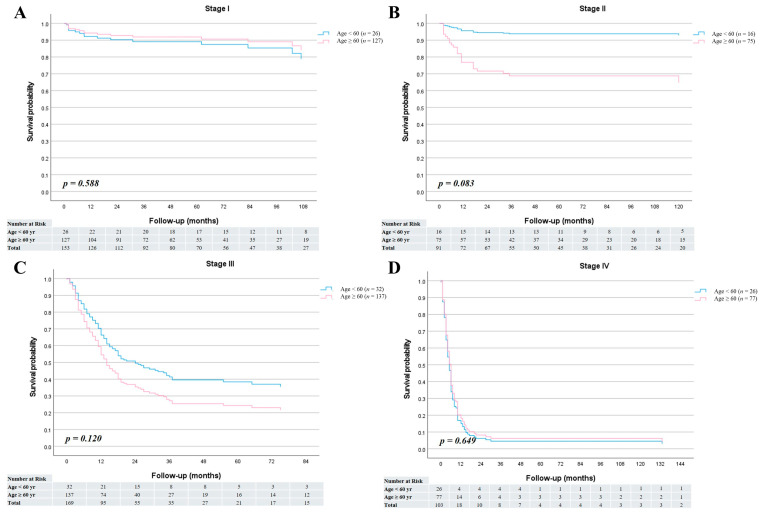
Stage-specific overall survival curves for patients aged <60 and ≥60 years. (**A**) Stage I (HR = 1.37; 95% CI, 0.44–4.27; *p* = 0.588). (**B**) Stage II (HR = 0.17; 95% CI, 0.02–1.26; *p* = 0.083). (**C**) Stage III (HR = 0.68; 95% CI, 0.41–1.11; *p* = 0.120). (**D**) Stage IV (HR = 1.11; 95% CI, 0.70–1.77; *p* = 0.649).

The median follow-up period was 30 months (range, 1–197 months) for patients younger than 60 years and 17 months (range, 1–188 months) for those aged 60 years or older. Among the survivors (*n* = 27 in the <60-year group and *n* = 232 in the ≥60-year group), the median follow-up durations were 67 months (range, 2–188 months) and 60 months (range, 3–197 months), respectively. In the overall cohort, no significant difference was observed in overall survival between the two age groups (HR = 0.92; 95% CI, 0.67–1.26; *p* = 0.593) (Figure 1A). Similarly, cancer-specific survival did not differ significantly between younger and older patients (HR = 0.87; 95% CI, 0.62–1.21; *p* = 0.408) (Figure 1B). The 1-, 3-, and 5-year overall survival rates were 62.9%, 53.1%, and 53.1% in the <60-year group, and 63.7%, 49.3%, and 48.2% in the ≥60-year group, respectively. Furthermore, stage-specific survival analysis demonstrated no statistically significant survival differences between the two age groups across all cancer stages (Figure 2).

## 4. Discussion

In this study, patients diagnosed with ESCC under the age of 60 showed a significantly stronger association with heavy alcohol consumption and smoking compared to older patients. Despite a higher prevalence of comorbidities and a greater proportion of patients who did not receive cancer-specific treatment among those aged ≥60 years, the overall survival between the two groups was similar. Moreover, patients in the <60-year group exhibited a higher tendency toward stage IV disease and distant metastasis at the time of diagnosis. These findings suggest that tobacco and alcohol use may contribute to the development of ESCC even at a relatively young age, and raise awareness that clinicians should not underestimate the potential severity of the disease in younger individuals. It is important for both the general public and healthcare providers to recognize that ESCC can occur in younger adults and may carry a similarly poor prognosis as in the elderly, underscoring the need for early detection and prompt treatment regardless of patient age.

Numerous epidemiological studies have demonstrated a strong association between ESCC and chronic exposure to alcohol and tobacco, which are considered the two most important modifiable risk factors for this malignancy [5,8]. Ethanol and acetaldehyde have been classified as group 1 carcinogens, particularly in relation to squamous epithelium of the esophagus [23]. Tobacco smoking independently increases the risk of ESCC through the inhalation and systemic circulation of nitrosamines, polycyclic aromatic hydrocarbons, and other genotoxic compounds [7]. A synergistic effect has been reported when both alcohol and smoking are present, leading to exponentially increased ESCC risk, especially in East Asian populations with genetic variants affecting alcohol metabolism [6,24]. In our study, patients with ESCC under the age of 60 had a significantly stronger association with heavy alcohol consumption and smoking compared to older patients. While few studies have directly compared risk factor profiles for ESCC by age, several have investigated the association between smoking or drinking patterns and ESCC risk. For smoking, longer duration of exposure was consistently associated with higher ESCC risk, suggesting that early initiation of smoking during youth may confer a particularly high risk of carcinogenesis in the esophageal epithelium [24,25]. Epidemiological evidence indicates that drinking patterns, specifically daily or binge drinking, are more strongly associated with an increased risk of ESCC than total alcohol consumption alone [26,27]. Mechanistically, high-concentration alcohol can more severely damage the esophageal mucosa, increasing permeability to carcinogens and facilitating DNA damage from metabolites such as acetaldehyde [27]. These findings provide supporting evidence that early and intense exposure to smoking and alcohol may predispose individuals to the earlier onset of ESCC. Other possible factors such as dietary habits, gastroesophageal reflux disease (GERD), and human papillomavirus (HPV) infection have also been investigated in previous studies. While GERD is more strongly associated with esophageal adenocarcinoma, chronic reflux-related mucosal inflammation may still contribute to squamous carcinogenesis in rare cases [5,28]. HPV infection has been identified in a minority of ESCC cases, although its etiologic role remains controversial [29,30,31]. In addition, one study from South Korea have reported a very low prevalence of high-risk HPV in ESCC, suggesting that HPV is unlikely to be a major contributing factor in Korean patients [32]. These variables were not consistently available in our retrospective dataset and were therefore not analyzed.

Multiple studies have shown that cancers occurring at a younger age may exhibit more aggressive biological behavior across various tumor types [14,15]. Esophageal cancer is no exception, with several studies reporting that younger patients are more likely to present with advanced-stage disease and distant metastases at diagnosis [13,33,34]. This tendency toward more advanced disease in younger patients may be related to more aggressive tumor biology or delayed diagnosis due to the lower clinical suspicion of cancer at a younger age. Although all of these studies consistently reported that younger patients presented with more advanced stages of disease, the survival outcomes varied. Compared to older patients, one study reported comparable overall survival in younger patients [34], another found shorter survival [33], and the most recent study showed improved survival among the younger cohort [13]. However, all of these investigations focused solely on esophageal adenocarcinoma. In contrast, our study exclusively examined patients with squamous cell carcinoma and found that individuals under the age of 60 had higher proportions of stage IV disease and distant metastasis at diagnosis, but demonstrated survival outcomes similar to those of older patients.

Several previous studies have examined age-related differences in outcomes following esophagectomy, with most using 70 years as the threshold to define elderly patients [35,36,37,38]. A meta-analysis demonstrated that patients aged 70 years or older had significantly higher in-hospital mortality and poorer 5-year overall survival compared to younger patients undergoing esophagectomy [38]. A large nationwide cohort study from Sweden also demonstrated that worse postoperative survival following esophagectomy began to appear from the age of 65–70 years and increased progressively in older age groups [36]. In our study, patients who underwent surgical resection were divided based on a 60-year age cutoff. There were no statistically significant differences in surgical outcomes between the two age groups. However, younger patients tended to have a lower postoperative mortality rate (3.3% vs. 8.6%, *p* = 0.190). Interestingly, the incidence of anastomotic stricture was higher in the younger group (20.0% vs. 13.2%, *p* = 0.207). This finding is somewhat unexpected, as known risk factors for anastomotic stricture such as diabetes mellitus (8.0% vs. 21.2%, *p* = 0.002) and anastomotic leakage (6.7% vs. 9.8%, *p* = 0.471) were both less common in younger patients. The role of age as a risk factor for anastomotic stricture after esophagectomy remains controversial, with existing studies showing inconsistent results [39]. Although the exact reason for the higher rate of anastomotic stricture in younger patients remains unclear, prior studies have indicated that excessive inflammation and fibrotic healing are major mechanisms of stricture formation [39,40]. While clear evidence is lacking, younger patients may exhibit a more vigorous inflammatory or wound-healing response, which could have contributed to this finding.

This study has several limitations. First, as a single-center retrospective study, the generalizability of the findings may be limited. Second, while heavy drinking was defined using internationally accepted criteria, individual variability in alcohol-related cancer risk could not be fully accounted for, and due to the retrospective nature of the study, the accuracy of self-reported alcohol consumption and smoking history may have been limited. Third, although patients under 60 years of age showed a trend toward higher rates of stage IV disease and distant metastasis at diagnosis, these differences did not reach statistical significance. Fourth, the shorter follow-up duration in the younger group may have affected survival comparisons, reflecting the smaller sample size and the retrospective nature of the study. Finally, because very few patients were younger than 50 years, we adopted 60 years as a practical cutoff for age stratification, which may not fully represent very early-onset ESCC but allowed meaningful subgroup analysis within our cohort.

## 5. Conclusions

In conclusion, patients with ESCC under the age of 60 demonstrated significantly stronger associations with heavy alcohol consumption and smoking compared to older patients. Despite their younger age and better overall performance status, they tended to present with more advanced disease and did not show better survival outcomes. These findings suggest that age itself may not be a decisive prognostic factor in ESCC. Future multicenter prospective studies are warranted to clarify the biological and clinical factors contributing to age-related differences and to develop tailored management strategies for younger patients with ESCC.

## Figures and Tables

**Table 1:** Baseline characteristics of all patients.

Characteristics	
**Total, *n***	516
**Age**	
median (range) years	69 (41–95)
<60 years, *n* (%)	100 (19.4)
**Sex, *n* (%)**	
male	464 (89.9)
female	52 (10.1)
**Past history, *n* (%)**	
Heavy drinking	235 (45.5)
Smoking	308 (59.7)
Gastrectomy	28 (5.4)
**Location, *n* (%)**	
Cervical	29 (5.6)
Upper thoracic	77 (14.9)
Mid-thoracic	235 (45.5)
Lower thoracic	175 (33.9)
* cStage, *n* (%)	
I	153 (29.7)
II	91 (17.6)
III	169 (32.8)
IV	103 (20.0)
**Initially distant metastasis, *n* (%)**	76 (14.7)
**ECOG performance status scale, *n* (%)**	
0, 1	298 (57.8)
2	68 (13.2)
3	141 (27.3)
4	9 (1.7)
**Comorbidities, *n* (%)**	
Hypertension	200 (38.8)
Diabetes	96 (18.6)
Cardiovascular disease	35 (6.8)
Cerebrovascular disease	37 (7.2)
Dementia	9 (1.7)
COPD	36 (7.0)
CKD	19 (3.7)
Cirrhosis	72 (14.0)
History of other malignancy	109 (21.1)
**Charlson Comorbidity Index, median (range)**	5 (2–13)
**Main treatment, *n* (%)**	
Surgery	234 (45.3)
Chemoradiotherapy or radiotherapy	108 (20.0)
Endoscopic resection	74 (14.3)
Chemotherapy	23 (4.5)
None	77 (14.9)

ECOG, Eastern Cooperative Oncology Group; COPD, Chronic Obstructive Pulmonary Disease; CKD, Chronic Kidney Disease. * Clinical tumor–node–metastasis (cTNM) according to the American Joint Committee on Cancer (AJCC) TNM version 8.

**Table 2:** Comparison of clinical characteristics and treatment patterns between patients aged <60 and ≥60 years.

	Age < 60 yr (*n* = 100)	Age ≥ 60 yr (*n* = 416)	*p* Value
**Age, median (range) years**	55 (41–59)	72 (60–95)	<0.001
**Sex, *n* (%)**			
Male	92 (92.0)	372 (89.4)	0.444
Female	8 (8.0)	44 (10.6)	
**Past history, *n* (%)**			
Heavy drinking	72 (72.0)	163 (39.2)	<0.001
Smoking	76 (76.0)	229 (55.0)	<0.001
Gastrectomy	1 (1.0)	27 (6.5)	0.060
**Location, *n* (%)**			
Cervical	4 (4.0)	25 (6.0)	0.437
Upper thoracic	14 (14.0)	63 (15.1)	0.773
Mid-thoracic	47 (47.0)	188 (45.2)	0.745
Lower thoracic	35 (35.0)	140 (33.7)	0.799
* cStage, *n* (%)			
I	26 (26.0)	127 (30.5)	0.374
II	16 (16.0)	75 (18.0)	0.633
III	32 (32.0)	137 (32.9)	0.858
IV	26 (26.0)	77 (18.5)	0.094
**Initially distant metastasis, *n* (%)**	18 (18.0)	58 (13.9)	0.305
**ECOG performance status scale, *n* (%)**			
0, 1	67 (67.0)	232 (55.8)	0.042
2	12 (12.0)	56 (13.5)	0.698
3	20 (20.0)	121 (29.1)	0.069
4	1 (1.0)	8 (1.9)	0.534
**Comorbidities, *n* (%)**			
Hypertension	19 (19.0)	181 (43.5)	<0.001
Diabetes	8 (8.0)	88 (21.2)	0.002
Cardiovascular disease	2 (2.0)	33 (7.9)	0.051
Cerebrovascular disease	3 (3.0)	34 (8.2)	0.085
Dementia	1 (1.0)	8 (1.9)	0.534
COPD	1 (1.0)	35 (8.4)	0.031
CKD	1 (1.0)	18 (4.3)	0.147
Cirrhosis	19 (19.0)	53 (12.7)	0.107
History of other malignancy	11 (11.0)	98 (23.6)	0.007
**Charlson Comorbidity Index, median (range)**	3 (2–11)	5 (4–13)	<0.001
**Main treatment, *n* (%)**			
Surgery	60 (60.0)	174 (41.8)	0.001
Chemoradiotherapy or radiotherapy	21 (21.0)	87 (20.9)	0.985
Endoscopic resection	6 (6.0)	68 (16.3)	0.011
Chemotherapy	3 (3.0)	20 (4.8)	0.436
None	10 (10.0)	67 (16.1)	0.128

ECOG, Eastern Cooperative Oncology Group; COPD, Chronic Obstructive Pulmonary Disease; CKD, Chronic Kidney Disease. * Clinical tumor–node–metastasis (cTNM) according to the American Joint Committee on Cancer (AJCC) TNM version 8.

**Table 3:** Surgical outcomes of patients aged <60 and ≥60 years.

	Age < 60 yr (*n* = 60)	Age ≥ 60 yr (*n* = 174)	*p* Value
**Age, median (range) years**	55 (48–59)	69 (60–90)	<0.001
**Gastrectomy history, *n* (%)**	1 (1.7)	10 (5.7)	0.227
**Location, *n* (%)**			
Cervical	1 (1.7)	1 (0.6)	0.449
Upper thoracic	5 (8.3)	29 (16.7)	0.122
Mid-thoracic	34 (56.7)	78 (44.8)	0.115
Lower thoracic	20 (33.3)	66 (37.9)	0.525
**Type of reconstruction**			
Esophago-gastric anastomosis	59 (98.3)	160 (92.0)	0.117
Esophago-colonic anastomosis	1 (1.7)	11 (6.3)	0.191
Esophago-jejunal anastomosis	0 (0)	3 (1.7)	0.552
**Neoadjuvant chemoradiotherapy**			
Yes, *n* (%)	11 (18.3)	40 (30.0)	0.441
Pathologic complete remission *, *n* (%)	3/11 (27.2)	12/40 (30.0)	0.861
**R0 resection, *n* (%)**	53 (88.3)	156 (89.7)	0.775
pStage **, *n* (%)			
0	3 (5.0)	12 (6.9)	0.607
I	18 (30.0)	49 (28.2)	0.786
II	12 (20.0)	42 (24.1)	0.513
III	22 (36.7)	63 (36.2)	0.949
IV	5 (8.3)	8 (4.6)	0.283
**Intraoperative mortality, *n* (%)**	0 (0)	0 (0)	0.597
**Postoperative mortality, *n* (%)**	2 (3.3)	15 (8.6)	0.190
**Anastomotic leakage, *n* (%)**	4 (6.7)	17 (9.8)	0.471
**Anastomotic stricture, *n* (%)**	12 (20.0)	23 (13.2)	0.207

* Absence of histologically identifiable residual cancer; ** Pathological stage after surgery.

## Data Availability

The data presented in this study are available on request from the corresponding author due to ethical reasons.

## Abbreviations

The following abbreviations are used in this manuscript:

ESCC	esophageal squamous cell carcinoma
ECOG	eastern cooperative oncology group
CCI	charlson comorbidity index
EUS	endoscopic ultrasound
CT	computed tomography
FDG	18Ffluorodeoxyglucose
PET	positron emission tomography
CRT	chemoradiotherapy
RT	radiation therapy
ESD	endoscopic submucosal dissection
HR	hazard ratio
CI	confidence interval
GERD	gastroesophageal reflux disease
HPV	human papillomavirus
